# Psychosocial developmental milestones of young adult survivors of childhood cancer

**DOI:** 10.1007/s00520-022-07113-3

**Published:** 2022-05-10

**Authors:** Heleen Maurice-Stam, Loes M. E. van Erp, Anne Maas, Hedy A. van Oers, Leontien C. M. Kremer, Eline van Dulmen-den Broeder, Wim J. E. Tissing, Jacqueline J. Loonen, Helena J. H. van der Pal, Laura R. Beek, Andrica C. H. de Vries, Marry M. van den Heuvel-Eibrink, Cécile M. Ronckers, Dorine Bresters, Marloes Louwerens, Margriet van der Heiden-van der Loo, Gea A. Huizinga, Martha A. Grootenhuis, on behalf of the Dutch LATER study group

**Affiliations:** 1 Princess Máxima Center for Pediatric Oncology, Heidelberglaan 25, 3584 CS Utrecht, The Netherlands; 2 Department of Child and Adolescent Psychiatry and Psychosocial Care, Amsterdam Reproduction and Development, Amsterdam Public Health, Emma Children’s Hospital, Amsterdam UMC, University of Amsterdam, Amsterdam, The Netherlands; 3 Department of Pediatrics, Amsterdam UMC, University of Amsterdam, Amsterdam, The Netherlands; 4 Amsterdam UMC/location VUmc, Amsterdam, The Netherlands; 5 Beatrix Children’s Hospital/University of Groningen/University Medical Center Groningen, Groningen, The Netherlands; 6 Radboud University Medical Center, Nijmegen, The Netherlands; 7 Sophia Children’s Hospital/Erasmus Medical Center, Rotterdam, The Netherlands; 8 Willem Alexander Children’s Hospital/Leiden University Medical Center, Leiden, The Netherlands; 9 Leiden University Medical Center, Leiden, The Netherlands

**Keywords:** Childhood cancer, Cancer survivors, Development, Psycho-oncology, Psychosocial functioning, Young adult

## Abstract

**Purpose:**

The study aimed to compare the psychosocial development of young adult survivors of childhood cancer (YACCS) with a norm group of young adults from the general population.

**Methods:**

From 2017 to 2020, 558 YACCS (18–30 years, 51% female, 10.9% CNS cancer) who participated in the Dutch Childhood Cancer Survivor Study (DCCSS) LATER cohort (diagnosed 1963–2001) part 2 completed the Course of Life Questionnaire (*CoLQ*), assessing the achievement of milestones. Items were grouped into the scales autonomy, psychosexual, and social development. Differences between YACCS and norm group were examined with ANOVA and Cohen’s *d* (*CoLQ* scales) and with logistic regression analysis and odds ratio (*OR*) (*CoLQ* items), for the total group and YACCS of CNS cancer.

**Results:**

The total group of YACCS did not report a less favorable psychosocial development than the norm group. YACCS of CNS cancer scored lower than the norm group (*p* < 0.001) on the scales autonomy (*d =* − 0.36) and psychosexual (*d* = − 0.46). Additionally, on half of the items of autonomy (0.25 *≤ OR ≤* 0.34), psychosexual (0.30 *≤ OR ≤* 0.48), and social (0.23 *≤ OR ≤* 0.47) development, YACCS of CNS cancer were less likely (*p* < 0.01) than the norm group to have achieved the milestones.

**Conclusion:**

Overall, psychosocial development of YACCS was as favorable as the norm, but YACCS of CNS cancer were at risk of an unfavorable psychosocial development in all domains. Monitoring psychosocial development should be included in the standards of psychosocial care, especially for CNS cancer patients and survivors, to be able to trace delay. Personalized interventions should be offered to improve the psychosocial development in an early stage.

## Background

Childhood cancer may have psychosocial consequences in the short and long term [1, 2]. To attain the goals in a typical psychosocial development, survivors of childhood cancer face extra challenges due to their disease history. Childhood cancer and its treatment often increase parental dependence and decrease participation in peer-based and school-based activities [3, 4]. Cognitive problems and non-attendance at school as a result of the disease and treatment can result in lowered educational achievement [5–7]. As a result, growing up with or after childhood cancer may have consequences for the psychosocial development of children, adolescents, and young adults.

The attainment of social and academic competence, peer relationships, independence from parents, and identity are generally recognized as important milestones in the development of a child into young adulthood [8–10]. The achievement of these psychosocial milestones is of importance to the adjustment in adult life [11–13]. Functioning of young adult childhood cancer survivors (YACCS) may be affected due to earlier missed experiences and delays in the achievement of psychosocial developmental milestones. Previous research in 2000/2001 among YACCS from the long-term follow-up clinic at the Academic Medical Center Amsterdam, The Netherlands [14], revealed that YACCS were at risk of a hampered psychosocial development. On group level, the differences with the general population were rather small [14], which indicated that the majority of YACCS were likely to have a favorable psychosocial developmental trajectory. However, YACCS of cancer in the central nervous system (CNS) and/or treated with radiotherapy appeared to be at risk of delays in the achievement of psychosocial developmental milestones, especially in the social and psychosexual domain [15]. Furthermore, YACCS who had achieved fewer psychosocial milestones while growing up were more likely to apply for disability benefits [16] and to experience worse health-related quality of life in young adulthood [15].

Care should not be limited to the physical and cognitive aspects of the disease but should also focus on the most optimal psychosocial functioning of the patient such as autonomy and social contacts with peers [17]. Knowledge about possible delay in the psychosocial development enables health care providers to aim for the most favorable psychosocial functioning of patients and survivors and to provide timely and relevant interventions. Literature about the achievement of psychosocial developmental milestones in survivors of childhood cancer is rather scarce. Since the first Dutch study 20 years ago [14], as far as we know, only a few, mostly small, studies about the psychosocial development of YACCS were published. Nies et al. [18] found no differences in psychosocial development between Dutch YACCS of childhood differentiated thyroid carcinoma and non-affected young adults, while Lehmann et al. [19], Van Dijk et al. [20], and Dieluweit et al. [21] demonstrated delay in psychosexual development in survivors of childhood cancer.

To expand the limited knowledge about the achievement of psychosocial developmental milestones while growing up with childhood cancer, the present study aimed to compare the psychosocial development of a nationwide cohort of YACCS with a norm group of young adults from the general population. We hypothesized that especially YACCS of CNS cancer achieved fewer psychosocial developmental milestones than the norm group.

## Methods

### Procedures and participants

#### YACCS from the Dutch Childhood Cancer Survivors LATER 2 Psycho-oncology study

Psycho-oncology data were collected between 2017 and 2020 as part of a nationwide cross-sectional cohort study: the Dutch Childhood Cancer Survivor Study (DCCSS) LATER cohort (diagnosed between 1/1/1963 and 31/12/2001) part 2, a clinical visit and questionnaire study. It concerned all patients diagnosed before the age of 18, and at least 5 years after diagnosis at time of study [22]. Survivors were included if they were living in The Netherlands at time of the childhood cancer diagnosis and treated in one of the seven former pediatric oncology/hematology centers in The Netherlands: Amsterdam University Medical Center (VU University Medical Center and Academic Medical Center Amsterdam), Leiden University Medical Center, Erasmus Medical Center Rotterdam, University Medical Center Groningen, Radboud University Medical Center Nijmegen, and University Medical Center Utrecht. Since 2018, pediatric oncology care in The Netherlands is centralized in the Princess Máxima Center for Pediatric Oncology in Utrecht.

YACCS, aged 18–30 years, who gave informed consent for the *DCCSS-LATER 2 Psycho-oncology study*, as part of the DCCSS-LATER 2 study, received a questionnaire about psychosocial developmental milestones (*Course of Life Questionnaire* (*CoLQ*)) at the end of their visit to the outpatient clinic for the DCCSS-LATER 2 study or by mail in case of no-show. YACCS had the opportunity to complete the questionnaire online or paper-pencil. Informed consent was obtained from all individual participants included in the study. The medical ethics boards of all participating centers approved the study (MEC2010_332).

#### Norm group of young adults

In 2012, 655 young adults from the general Dutch population, aged 18–30 years, completed the *CoLQ* to update previous normative data of the *CoLQ* that were collected in the context of research among YACCS in 2000/2001 [14]. Data were collected online in cooperation with TNS NIPO (operating under the name of “Kantar Public”), a Dutch market research agency. A stratified sample was drawn from a panel of TNS NIPO, based on Dutch population figures regarding key demographics (age, sex, marital status, and education) [23].

### Measures

The CoLQ was used to assess the achievement of psychosocial developmental milestones [14]. The CoLQ was developed, validated, and normed in The Netherlands in 2000–2001 [14, 24] and updated in 2012 (see “Procedures and participants”). In the meantime the *CoLQ* was used in almost 2000 young adults grown up with 18 different pediatric diseases [25]. The CoLQ asks retrospectively whether, or at what age, the respondent had achieved certain milestones. It concerns behaviors that are characteristic of certain age stages, developmental tasks, or limitations children might encounter when they grow up with a chronic disease. The items (milestones) and answer categories, such as the age at which a certain milestone is expected to be achieved in the majority of children and adolescents, were based on the literature and clinical experience of developmental psychologists.

The items are divided into five domains: three psychosocial developmental domains and two risk behavior domains. In the present study, the items of the three psychosocial domains were used: autonomy development (6 items about autonomy at home and outside home; scale score range 6–12), psychosexual development (4 items about love and sexual relations; scale score range 4–8), social development (12 items about social contacts with peers at school and in leisure time; scale score range 12–24). A higher score indicates the accomplishment of more psychosocial developmental milestones. Apart from the scale scores, the individual items can be used as outcomes because each item represents a milestone.

Validity, test-retest reliability, and internal consistency of the *CoLQ* were satisfactory in previous studies, though the internal consistency of autonomy was moderate [14, 15, 24]. Cronbach’s alphas in the present study were as follows for autonomy, psychosexual, and social development, respectively: norm group 0.49, 0.77, and 0.74; YACCS 0.54, 0.79, and 0.76.

#### Socio-demographic and medical characteristics

Data on age, sex, and medical characteristics (see Table 1

**Table 1:** Demographic and medical characteristics of YACCS: participants versus non-participants/norm group

	Participants (*N* = 558)			Non-participants (*N* = 858)^a^				Norm group (*N* = 655)^b^			
	*M*	SD	Range	*M*	SD	Range	*p*	*M*	SD	Range	*p*
*Age at study* (years)	25.78	3.33	18.10–30.97	25.48	3.45	18.01–31.00	0.10	24.75	3.79	18.01–30.99	**< 0.001**
*Age at first diagnosis* (years)	4.25	3.12	0.00–14.67	4.31	2.99	0.00–14.62	0.73				
*Time since first diagnosis* (years)	20.92	3.31	15.02–29.17	21.18	3.42	14.81–30.93	0.16				
	*N*		%	*N*		%	*p*	*N*		*%*	*p*
*Sex*							**< 0.001**				0.977
Male	273		48.9	530		61.8		321		49.0	
Female	285		51.1	328		38.2		334		51.0	
*Age at first diagnosis* (years)							0.86				
0–5	418		74.9	644		75.1					
6–11	128		22.9	199		23.2					
12–15	12		2.2	15		1.7					
*Time since first diagnosis* (years)							0.19				
10–19	258		46.2	375		43.7					
20–29	300		53.8	479		55.8					
30–39	0		0.0	4		0.5					
*Diagnosis*^c^							0.76				
Leukemia/lymphoma	299		53.6	453		52.8					
Solid tumor	198		35.5	300		35.0					
CNS cancer^*d*^	61		10.9	105		12.2					
*Treatment*^e^							0.10				
Chemotherapy only (with/without surgery)	394		70.6	585		68.3					
Radiotherapy only (with/without surgery)	14		2.5	33		3.9					
Surgery alone	48		8.6	96		11.2					
Combination therapy (chemo + radiotherapy, with/without surgery)	98		17.6	129		15.1					
No treatment	4		0.7	14		1.6					
*Hematopoietic transplant*							**0.03**				
Yes	46		8.3	46		5.4					
No	511		91.7	811		94.6					
*Relapse*							0.63				
Yes	61		10.9	101		11.8					
No	497		89.1	757		88.2					

Significant differences between participants and non-participants and between participants and norm group are presented in bold

^a^YACCS who did not participate in the present study or any other part of the *DCCS-LATER study part 2*

^b^The norm group consists of young adults from the general Dutch population

^c^First, primary diagnosis

^d^Central nervous system and miscellaneous intracranial and intraspinal neoplasm

^e^For primary cancers and recurrences

### Statistical analyses

Differences between participants and non-participants/norm group were tested with independent *t* tests and chi-square tests. Analysis of variance (ANOVA) by group, age, and sex was performed to test differences between YACCS and the norm group on the mean scale scores of the *CoLQ*. Effect sizes *d* were calculated by dividing the difference in mean scores between YACCS and the norm group by the standard deviation in the norm group. After Cohen [26], effect sizes up to 0.2 were considered to be small, effect sizes about 0.5 to be medium, and effect sizes of about 0.8 to be large. Because the distribution of the scale scores of psychosexual development and social development was left skewed, we checked the results with non-parametric tests (Mann-Whitney *U* tests).

In order to gain more detailed insight into the psychosocial development, differences between YACCS and the norm group on item level, indicating the achievement of individual milestones, were explored additionally. Logistic regression analyses were carried out with the achievement of a milestone (yes/no) as dependent variable and group (YACCS versus the norm group), age, and sex as independent variables, including odds ratios (ORs).

The analyses were conducted for the total group of YACCS and for YACCS of CNS cancer. A significance level of 0.013 was used for the analyses on scale level: 0.05 divided by the number of three scales. For the explorative analyses on item level, a significance level of 0.01 was used.

## Results

### Participants

Of the 1416 eligible YACCS in the age range 18–30 years, a total of 828 (58.5%) participated in the DCSS-LATER 2 study. The most frequent reasons for non-participation were deceased, lost to follow-up, living abroad, and refusal. Of the 828 YACCS who participated in the DCSS-LATER 2 study, 558 (67.4%) participated also in the *DCCSS-LATER 2 Psycho-oncology study* and completed the *CoLQ*. Reasons for refusal and non-response to the *DCCSS-LATER 2 Psycho-oncology study* were not administrated. The percentages of female sex and hematopoietic transplant were significantly higher in participants from the present study (*CoLQ*) than in YACCS who did not participate in the present study or other parts of the DCCSS-LATER 2 study (51.1% versus 38.2% and 8.3% versus 5.4%, respectively).

The total group of YACCS was older than the norm group (mean 25.78, SD 3.33 versus mean 24.75, SD 3.79; *p* 0.00) but they did not differ on sex (51.1% versus 51.0% female). YACCS of CNS cancer (mean 26.78, SD 3.35; *p* 0.00) were also older than the norm group and the proportion of female was higher in YACCS of CNS cancer than in the norm group (68.9% vs 51.0%, *p* 0.01).

### Psychosocial developmental milestones on scale level

#### YACCS total group versus the norm group

No significant differences (*p* < 0.013) were found between the *CoLQ* mean scale scores of the total group of YACCS and the norm group according to ANOVA by group, age, and sex (Table 2). Mann-Whitney *U*

**Table 2:** Psychosocial developmental milestones YACCS (total group and CNS cancer) versus the norm group; *CoLQ* scale scores^a^

	*YACCS total group*			*YACCS CNS cancer*			*Norm group*			*YACCS total vs norm*	*CNS cancer vs norm*
	Males	Females	Total	Males	Females	Total	Males	Females	Total	Effect size *d*	Effect size *d*
**Autonomy**										0.05	**− 0.36**
*Mean*	9.17	9.17	9.17	8.63	8.52	**8.56** ^b^*	8.95	9.22	9.09		
*SD*	1.52	1.55	1.53	1.64	1.55	1.57	1.44	1.46	1.46		
***N***	272	282	554	19	42	61	321	334	655		
**Psychosexual**										0.06	**− 0.46**
*Mean*	6.87	6.90	6.88	6.33	6.10	**6.17** ^b^*	6.64	6.94	6.80		
*SD*	1.35	1.39	1.37	1.57	1.38	1.43	1.38	1.35	1.37		
***N***	267	280	547	18	42	60	321	334	655		
**Social**										0.15	**−** 0.26
*Mean*	20.92	20.35	20.63	20.47	19.12	19.51	20.12	20.33	20.23		
*SD*	2.67	2.83	2.76	2.58	2.81	2.79	2.89	2.59	2.74		
***N***	264	279	543	17	42	59	321	334	655		

*CNS* central nervous system and miscellaneous intracranial and intraspinal neoplasm

Cohen’s *d*: mean score of *YACCS total group* or *YACCS CNS cancer* minus the mean score of the norm group divided by the standard deviation of the norm group (according to Cohen [26]). After Cohen [26], effect sizes up to 0.2 were considered to be small, effect sizes about 0.5 were considered to be medium, and effect sizes of about 0.8 were considered to be large

*p < 0.001

Significant differences (*p* < 0.013) between YACCS and the norm group are presented in bold

^a^A higher scale score indicates that more milestones were achieved

^b^*YACCS of CNS cancer* scored significantly lower than the norm group, according to ANOVA corrected for age and sex

#### YACCS of CNS cancer versus the norm group

CNS YACCS had significantly lower mean *CoLQ* scale scores than the norm group on autonomy development (*d* − 0.36; *p <* 0.001) and psychosexual development (*d* − 0.46; *p* < 0.001). YACCS of CNS cancer did not differ significantly from the norm group on social development (*d* − 0.26; *p* 0.035) (Table 2). Mann-Whitney *U* tests yielded similar results.

### Psychosocial developmental milestones on item level

#### YACCS total group versus the norm group

The total group of YACCS did not differ significantly (*p* < 0.01) from the norm group on the items of autonomy and psychosexual development (Table 3). In the social development domain, the total group of YACCS was significantly more likely to have been member of a sports club, in the period of secondary school (*OR* 1.80, *p <* 0.001) and after secondary school (*OR* 1.72, *p*

**Table 3:** Psychosocial developmental milestones YACCS (total group and CNS cancer) versus the norm group; *CoLQ* item scores

	YACCS total group		YACCS CNS cancer		Norm group		Total vs Norm	CNS cancer vs norm
	%	*N*	%	*N*	%	*N*	*OR* [99% CI]	*OR* [99% CI]
	*(a) Frequencies of the (dichotomized) items of* ***autonomy development***							
Regular chore in your family, e.g., washing dishes, setting the table; primary school								
*Yes* *No*	33.4 66.6	186 371	29.5 70.5	18 43	39.5 60.5	259 396	0.74 [0.54; 1.01]	0.57 [0.27; 1.22]
Paid jobs/chores outside home, e.g., washing cars, mowing lawns; primary school								
*Yes* *No*	31.7 68.3	177 381	23.0 77.0	14 47	23.8 76.2	156 499	1.39 [0.99; 1.94]	0.81 [0.35; 1.86]
Regular job/chore in your family, secondary school								
*Yes* *No*	55.3 44.7	308 249	54.1 45.9	33 28	52.2 47.8	342 313	1.14 [0.84; 1.54]	1.03 [0.51; 2.08]
Paid jobs, secondary school								
*At the age of 18 or younger* *At the age of 19 or older/never*	81.5 18.5	455 103	62.3 37.7	38 23	85.0 15.0	557 98	0.76 [0.51; 1.14]	**0.27**^a**^ [0.13; 0.57]
For the first time being on holiday without adults								
*At the age of 17 or younger* *At the age of 18 or older/never*	44.4 55.6	247 309	21.3 78.7	13 48	42.6 57.4	279 376	1.05 [0.78; 1.42]	**0.34**^a*^ [0.15; 0.78]
Leaving your parents’ place								
*Not living with your parents* *Still living with your parents*	69.7 30.3	388 169	65.6 34.4	40 21	65.8 34.2	431 224	0.70 [0.47; 1.06]	**0.25**^a**^ [0.09; 0.65]
	*(b) Frequencies of the (dichotomized) items of* ***psychosexual development***							
First girlfriend/boyfriend								
*At the age of 17 or younger* *At the age of 18 or older/never*	64.5 35.5	359 198	45.9 54.1	28 33	63.4 36.6	415 240	1.00 [0.73; 1.37]	**0.44**^a*^ [0.22; 0.89]
For the first time falling in love								
*At the age of 18 or younger* *At the age of 19 or older/never*	88.8 11.2	492 62	78.7 21.3	48 13	86.6 13.4	567 88	1.16 [0.73; 1.83]	0.51 [0.21; 1.23]
For the first time sexual intimacy								
*At the age of 18 or younger* *At the age of 19 or older/never*	75.3 24.7	417 137	60.0 40.0	36 24	72.4 27.6	474 181	1.10 [.78; 1.55]	**0.48**^a*^ [0.23; 0.99]
For the first time sexual intercourse								
*At the age of 18 or younger* *At the age of 19 or older/never*	59.5 40.5	328 223	32.8 67.2	20 41	57.4 42.6	376 279	1.04 [0.77; 1.42]	**0.30**^a**^ [0.14; 0.63]
	*(c) Frequencies of the (dichotomized) items of* ***social development***							
At least one year of membership in a sports club, primary school								
*Yes* *No*	84.5 15.5	469 86	91.7 8.3	55 5	80.3 19.7	526 129	1.37 [0.92; 2.04]	2.83 [0.82; 9.75]
Number of friends in first–third grade, primary school								
*4 or more* *Less than 4*	61.0 39.0	340 217	50.8 49.2	31 30	60.0 40.0	393 262	1.04 [0.76; 1.41]	0.67 [0.33; 1.34]
Number of friends in fourth–sixth grade, primary school								
*4 or more* *Less than 4*	63.4 36.6	352 203	50.8 49.2	31 30	60.3 39.7	395 260	1.14 [0.84; 1.56]	0.68 [0.34; 1.38]
Best friend, primary school								
*Yes* *No*	78.7 21.3	437 118	70.5 29.5	43 18	71.9 28.1	471 184	1.39 [0.97; 1.97]	0.81 [0.37; 1.75]
Most of the time playing with …, primary school								
*Friends* *Brothers and/or sisters, parents, on your own*	81.5 18.5	455 103	72.1 27.9	44 17	84.0 16.0	550 105	0.83 [0.56; 1.23]	0.46 [0.21; 1.02]
At least one year of membership in a sports club, secondary school								
*Yes* *No*	74.5 25.5	415 142	81.7 18.3	49 11	62.4 37.6	409 246	**1.80**^b**^ [1.29; 2.50]	**2.81**^b*^ [1.15; 6.85]
Number of friends, secondary school								
*4 or more* *Less than 4*	64.5 35.5	360 198	42.6 57.4	26 35	62.0 38.0	406 249	1.15 [0.84; 1.57]	**0.46**^a*^ [0.23; 0.94]
Best friend, secondary school								
*Yes* *No*	70.6 29.4	393 164	63.9 36.1	39 22	66.1 33.9	433 222	1.22 [0.88; 1.69]	0.79 [0.38; 1.66]
Belonging to a group of friends, secondary school								
*Yes* *No*	80.1 19.9	444 110	57.4 42.6	35 26	78.6 21.4	515 140	1.14 [0.79; 1.66]	**0.37**^a**^ [0.18; 0.76]
Leisure time, mainly with …, secondary school								
*Friends* *Brothers and/or sisters, parents, on your own*	79.9 20.1	446 112	57.4 42.6	35 26	83.5 16.5	547 108	0.74 [0.50; 1.10]	**0.23**^a**^ [0.11; 0.48]
Going out to a bar or disco, secondary school								
*Sometimes/often* *Never*	74.4 25.6	415 143	60.7 39.3	37 24	74.0 26.0	485 170	0.93 [0.66; 1.32]	**0.47**^a*^ [0.23; 0.99]
At least one year of membership in a sports club, after secondary school								
*Yes* *No*	53.0 47.0	296 262	57.4 42.6	35 26	39.4 60.6	258 397	**1.72**^b**^ [1.27; 2.34]	**2.22**^b*^ [1.09; 4.52]

*CNS* central nervous system and miscellaneous intracranial and intraspinal neoplasm; *OR* odds ratio/exponent B; *CI* confidence interval

*p < 0.01; **p < 0.001; significant differences (*p* < 0.01) between YACCS and the norm group are presented in bold

^a^The proportion of *YACCS CNS cancer* that achieved the milestone was significantly lower than the proportion in the norm group, according to logistic regression analyses corrected for age and sex

^b^The proportion of *YACCS CNS cancer* that achieved the milestone was significantly higher than the proportion in the norm group, according to logistic regression analyses corrected for age and sex

#### YACCS of CNS cancer versus the norm group

In the autonomy development domain, YACCS of CNS cancer were significantly less likely than the norm group to have achieved three out of the six milestones (Table 3). It concerned having a paid job in the period of secondary school (*OR* 0.27, *p* < 0.001), going on holiday without adults before the age of 18 (*OR* 0.34, *p* 0.001), and leaving their parents’ place (*OR* 0.25, *p <* 0.001).

With regard to psychosexual development, YACCS of CNS cancer were significantly less likely than the norm group to have achieved three out of the four milestones: first girlfriend or boyfriend before the age of 18 (*OR* 0.44, *p* 0.003), for the first time sexual intimacy before the age of 19 (*OR* 0.48, *p* 0.009), for the first time sexual intercourse before the age of 19 (*OR* 0.30, *p* < 0.001).

In the social development domain, YACCS of CNS cancer were significantly less likely than the norm group to have achieved the following four milestones out of twelve: having more than four friends (*OR* 0.46, *p* 0.005), belonging to a group of friends (*OR* 0.37, *p* < 0.001), spending leisure time with friends (*OR* 0.23, *p* < 0.001), and going out to a bar or disco (*OR* 0.47, *p* 0.009), in the period of secondary school. They were significantly more likely to have been members of a sports club: in the period of secondary school (*OR* 2.81, *p* 0.003) and after secondary school (*OR* 2.22, *p* 0.004).

## Discussion

Overall, the psychosocial development of the total group of YACCS was as favorable as the psychosocial development of peers from the general Dutch population, while YACCS of CNS cancer appeared to be at risk of an unfavorable psychosocial development. YACCS of CNS cancer achieved half as many milestones as their peers in all three psychosocial developmental domains with differences on scale scores of small-to-moderate size. On the positive side, they were more likely to have been members of a sports club, which is in favor of their social contacts with peers, apart from the physical health advances. This positive result was also found in the total group of YACCS.

On the one hand, the results were not surprising because it is generally known from previous research that, overall, survivors of childhood cancer function well psychosocially, while problems were seen in subgroups of survivors [27], especially in survivors of CNS cancer [1, 15, 28, 29]. The cognitive problems many survivors of CNS cancer face could increase dependence from parents and complicate contacts with peers, which in turn could result in delay of the achievement of psychosocial developmental milestones.

On the other hand, the favorable psychosocial development of the total group of YACCS was not expected because the psychosocial development of YACCS appeared to be hampered in a previous study, 20 years ago [14]. The explanation of these conflicting results is probably twofold. First, improvements in treatment, efforts to reduce toxicity of treatment in particular, and improvements in (psychosocial) care over the past 15 to 20 years may have helped prevent adverse consequences for psychosocial development. Overall, YACCS in the present study tend to have higher scores on the scales and items of the *CoLQ* than the YACCS 20 years ago, which indicates that the psychosocial development of YACCS improved between 2000 and 2020. A second explanation may lie in the normative data that reflect developments in Dutch society. In the current normative data, several milestones were achieved by a lower proportion of young adults than in the normative data from 2000 to 2001 [14], for example, membership of sports clubs and age at first sexual intercourse. This is in line with developments in the Dutch society [30, 31] but these developments were not seen in survivors. Maybe this kind of societal developments had less impact on children and adolescents whose life was all about surviving and dealing with the consequences of childhood cancer and its treatment. Regarding the result that survivors were more likely to have been member of a sports club than their peers, it could also be that patients and survivors of childhood cancer were more strongly stimulated to participate in sport clubs than children and adolescents without a history of childhood cancer. It is likely that health care providers and parents more and more focus on improving quality of life and well-being and consider participation in a sports club an effective way to improve physical and social well-being. The explanations discussed above contribute to smaller differences between the psychosocial development of YACCS and peers, in favor of the YACCS as total group.

### Study limitations

These results yield insight into the psychosocial development of a large nationwide cohort of YACCS but the results do not paint the complete picture. The psychosocial development is more comprehensive than the milestones assessed retrospectively with the *CoLQ*. To prevent recall bias, the milestones were strictly factual and do not go further back than the period of primary school. Another limitation of the *CoLQ* concerns the moderate internal consistency of the autonomy development scale. It is acceptable to use scales with moderate internal consistency for group comparisons because internal consistency gives an indication of random error; it has nothing to do with systematic error. However, larger random errors make it more difficult to detect differences between groups [32]. This limitation was partly overcome by the analysis of the individual milestones within the scales.

Although more than two-thirds of the YACCS who participated in the DCCSS-LATER 2 study completed the *CoLQ*, the overall response rate was moderate. Probably this did not affect the representativeness of the sample because the medical history of the participants and non-participants was very similar. It is unlikely that the difference in hematopoietic transplant between participants and non-participants biased the results because the number of YACCS who received hematopoietic transplant was small. Finally, the representativeness of the Dutch norm group was not optimal. Our norm sample was more often born in The Netherlands than the general Dutch population (97% versus 91%), more often highly educated (30% versus 25%), more often employed (69% versus 59%), and more often married or living together with a partner (36% versus 31%) [33]. However, we can only speculate about the possible confounding effect on the results of our study because three out of these demographics (educational level, employment, and marital status) can be considered a possibly affected outcome of being a survivor of childhood cancer [14, 34, 35].

The present study was focused on psychosocial development in survivors of childhood cancer as an outcome. Apart from a diagnosis of CNS cancer, medical risk factors of an unfavorable psychosocial development were not addressed. Future research should focus on this.

### Clinical implications

Since most of the children and adolescents with cancer reach adulthood today, health care providers need to understand the psychosocial consequences of growing up with or after childhood cancer. Knowledge about possible delay in the psychosocial development of patients and survivors could help optimizing their development to adulthood and achieving a sustainably good quality of life in adulthood. Though overall survivors in this study showed no delay in psychosocial development, survivors of CNS cancer appeared to be at risk of a suboptimal psychosocial development. Further research should reveal whether other subgroups at risk could be designated.

Attention to the achievement of psychosocial milestones is warranted to detect and support those at risk at an early stage. It is recommended to include monitoring of psychosocial developmental milestones in the standards of psychosocial care for patients and survivors [36, 37], especially for patients and survivors of CNS cancer, and especially at important transition moments such as the transition from primary to secondary school or the transition from school to work. Monitoring should not stop after transition from pediatric to adult health care because survivors who were delayed in their psychosocial development deserve attention and support into adulthood. Follow-up of survivors is also important because “growing into deficit” is a known phenomenon, especially in survivors with cognitive late effects of diagnosis and treatment [38]. Monitoring can be facilitated by electronic systems that assess patient-reported outcomes, for example, the Dutch KLIK-PROM system [39].

Interventions to optimize psychosocial development should focus on changeable, psychosocial factors, such as coping with the consequences of childhood cancer by patients, survivors, and parents. Parents and other caregivers should be encouraged to stimulate autonomy by treating patients and survivors as normally as possible and avoid overprotection. In addition to stimulating autonomy in daily life, it is important to empower survivors to take control of their own health. Stimulating patients and survivors to join in activities with peers is important for their psychosocial functioning. Group programs based on cognitive behavioral-based therapy (CBT) [40] or acceptance and commitment therapy (ACT) [41] could be helpful in stimulating coping with the consequences of childhood cancer and could prevent and diminish psychosocial problems in patients, survivors, and parents [42, 43].

We found that especially survivors of CNS cancer were less likely to have a paid job during adolescence; while jobs during adolescence increase the likelihood of job participation in adulthood [16], offer the possibility to gain work experience and to earn own money, and improve self-esteem. Therefore, it is recommended to support adolescents in finding (paid) jobs. Last but not least, a personalized approach is of utmost importance, especially in case of CNS cancer because of the complex and individual consequences of CNS cancer. It is important to find out which psychosocial milestones are feasible within the capabilities of the patient or survivor. Care providers who are familiar with the consequences of childhood cancer in general and with the history of the patient or survivor in particular could be helpful in setting goals that are meaningful and achievable taking into account the personal beliefs and growth of the patient or survivor.

## Conclusions

Overall, the psychosocial development of survivors was as favorable as in the general population, but survivors of CNS cancer appeared to be at risk of an unfavorable development in all three developmental domains: autonomy, psychosexual, and social development. Monitoring of the achievement of psychosocial development should be included in the standards of psychosocial care especially for CNS cancer patients and survivors in order to be able to trace and minimize delay in the psychosocial development at an early stage. Electronic systems that assess patient-reported outcomes can be used to trace patients in need for specific support. Considering the complex and individual consequences of CNS cancer, especially CNS cancer survivors need a personalized approach.

## Data Availability

The datasets generated and/or analyzed during the current study are available from the corresponding author on reasonable request.
